# Supplementary material for: In situ study on atomic mechanism of melting and freezing of single bismuth nanoparticles
Source: Nat Commun. 2017 Feb 13;8:14462. doi: 10.1038/ncomms14462 (PMC5316836; doi:10.1038/ncomms14462)
Supplement: Supplementary Information — Supplementary Figures and Supplementary Reference. [file ncomms14462-s1.pdf]

Supplementary Figures

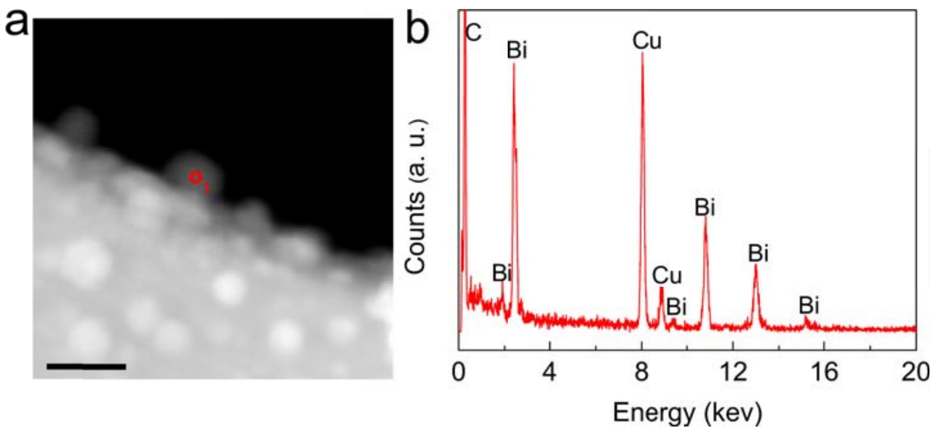

Supplementary Figure 1. a, The image obtained by high-angle annular dark-field scanning transmission electron microscopy (HAADF-STEM). **b**, EDS analysis of a single Bi nanoparticle anchored to the edge of $SrBi_2Ta_2O_9$ platelet, as marked by the red circled region in **a**. The EDS result clearly reveals that the nanoparticle is composed of elemental Bi. The scale bar in **a** is 20 nm.

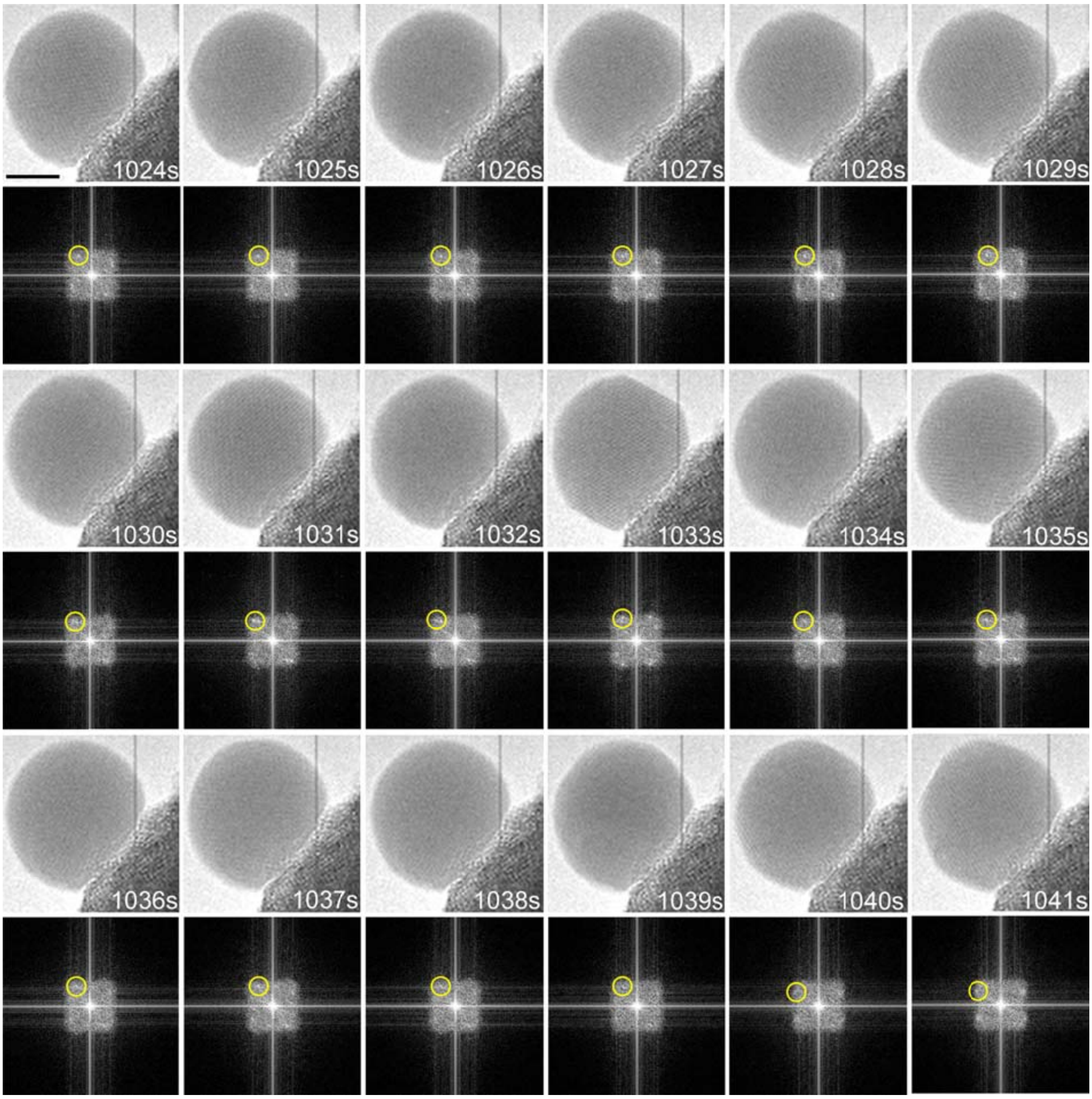

Supplementary Figure 2. Sequential snapshots of HRTEM imaging showing the long-range correlation in the nanoparticle. The images are sampled every second between 1024 and 1041s from Supplementary Movie 3. The FFT pattern for each HRTEM image appears beneath the image. The scale bar in **a** is 5 nm. The scale bar applies to all the HRTEM images.

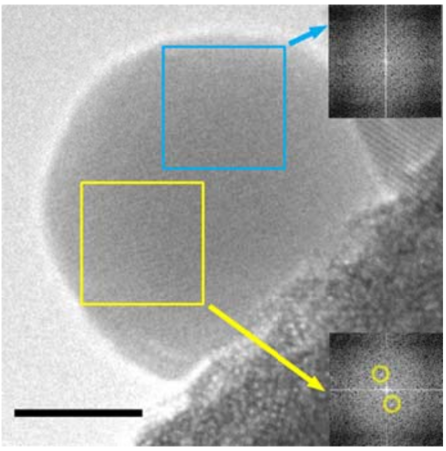

Supplementary Figure 3. Still HRTEM image of the Bi nanodroplet recorded after the 1344 s period of video. The inset shows the corresponding FFT patterns of the regions as marked by the blue and yellow squares. The uniform contrast of the HRTEM image and the FFT pattern confirm the liquid nature of the overall nanoparticle. FFT analysis of the region marked by the yellow square shows diffraction spots (marked by yellow circles) that could be associated with short-range crystalline order, indicating the existence of small crystalline embryo in the liquid regime. The scale bar is 5 nm.

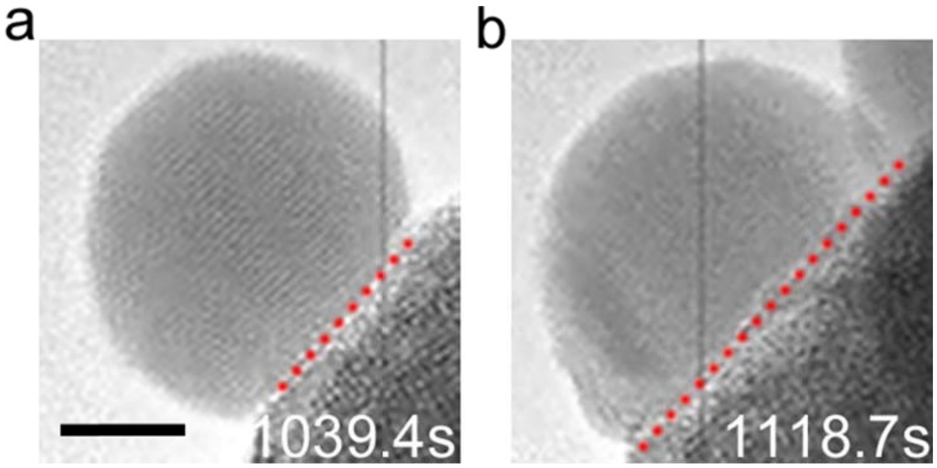

Supplementary Figure 4. a, **b**, The contacts between the Bi nanodroplet and the support before and after the first crystallization of the nanodroplet at 1099.7 s. The lengths of contact lines are highlighted by the dashed red lines in the HRTEM images. The contact areas are approximately regarded as circular with diameters of 8.4 nm and 14.8 nm, respectively. The contact areas were calculated to be 55.4 nm^2 and 117.9 nm^2, respectively. It is clear that the contact area is greatly increased after the first crystallization of the nanodroplet. The scale bar in **a** is 5 nm. The scale bar applies to all images.

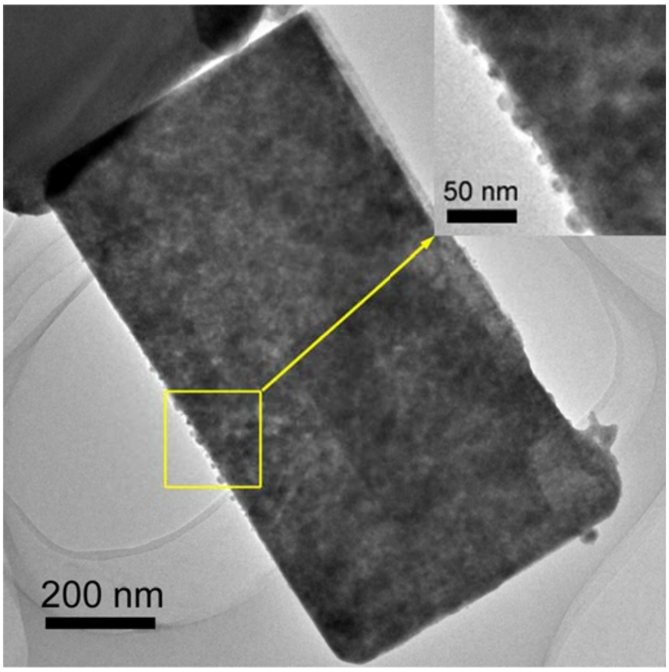

Supplementary Figure 5. TEM image of $SrBi_2Ta_2O_9$ sample after 40 h photoreaction. Photoreaction was performed at 25 °C under atmospheric pressure in a closed circulation system using a high-pressure Hg lamp (500 W). A water filter was used to remove infrared light. $SrBi_2Ta_2O_9$ powder (0.2 g) was dispersed and magnetically stirred in a Pyrex top-irradiation reaction cell containing 90 mL H_2O and 15g $C_6H_{12}O_6 \cdot H_2O$. The suspension was evacuated prior to irradiation and was maintained at about 25 °C by immersing the reaction cell in a water bath with a constantly controlled temperature.

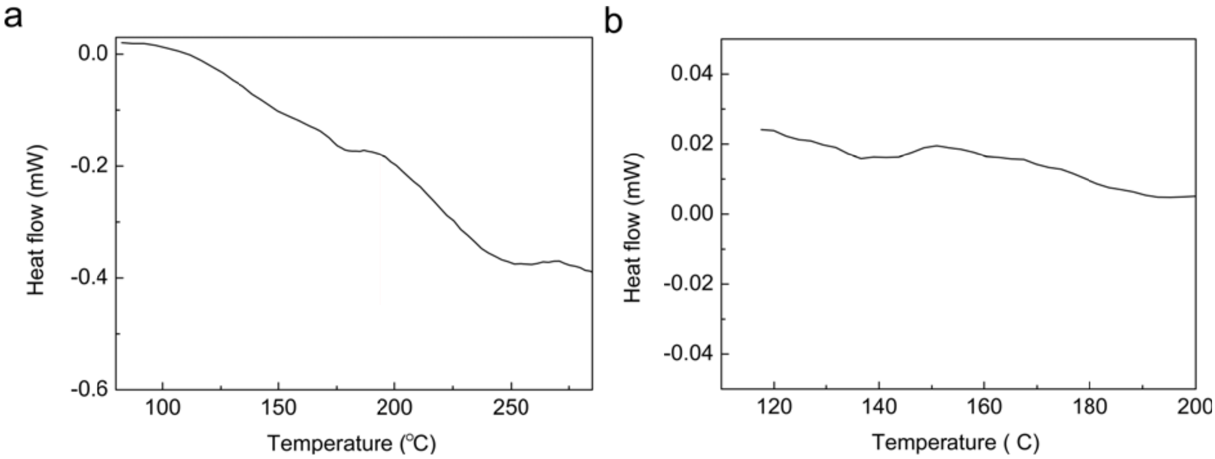

Supplementary Figure 6. a, **b**, The heating and cooling DSC curves of the Bi nanoparticles synthesized by a photoreaction method.

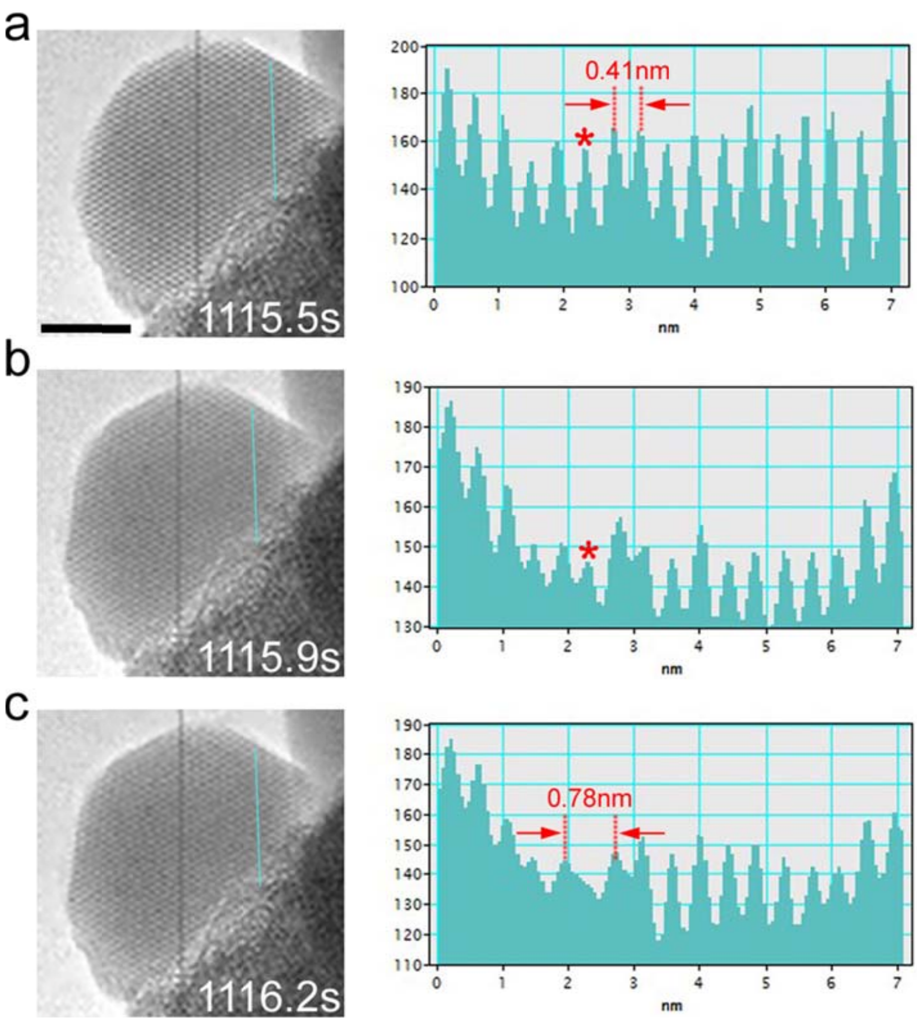

Supplementary Figure 7. a-c, The intensity profiles recorded along the blue line in HRTEM image of Figs 3a-c. The intensity profiles clearly feature contrast variations along the lines. Each peak in the intensity profiles represents a corresponding atomic column in the HRTEM images. The distance between adjacent atomic columns along the blue line in HRTEM image of **a** is about 0.41 nm. The intensity of the peak marked by the red asterisk decreased with time. This peak disappears at 1116.2 s as shown in **c**, indicating the formation of a vacancy in the position of Bi column marked by the red asterisk. As a result, a gap of 0.78 nm between adjacent atomic columns is formed as shown in **c**, which is about two times of 0.41 nm in **a**. In comparison, as shown in **c**, the distance of the other adjacent atomic columns along the blue line did not change significantly. The scale bar in **a** is 5 nm. The scale bar applies to all HRTEM images.

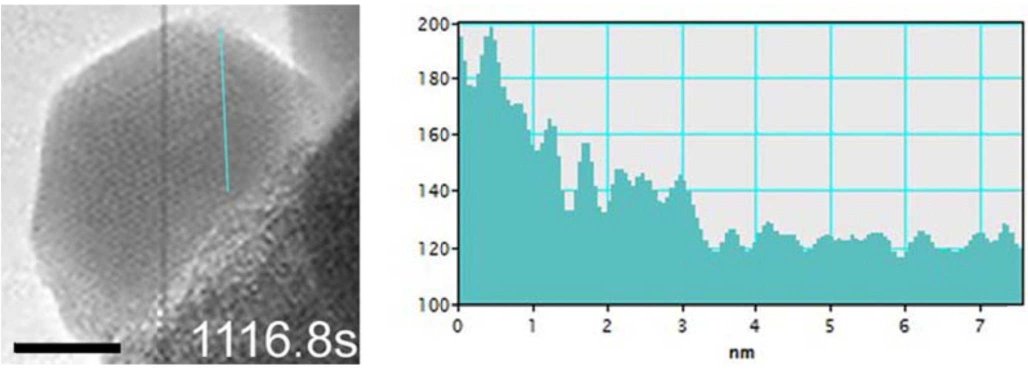

Supplementary Figure 8. The intensity profile recorded along the blue line in HRTEM image of Fig. 2e. The intensity profile clearly features contrast variation along the line. The weak peaks between 4 and 7.5 nm are due to weak contrast variations along the line, which is a characteristic of liquid phase. The scale bar is 5 nm.

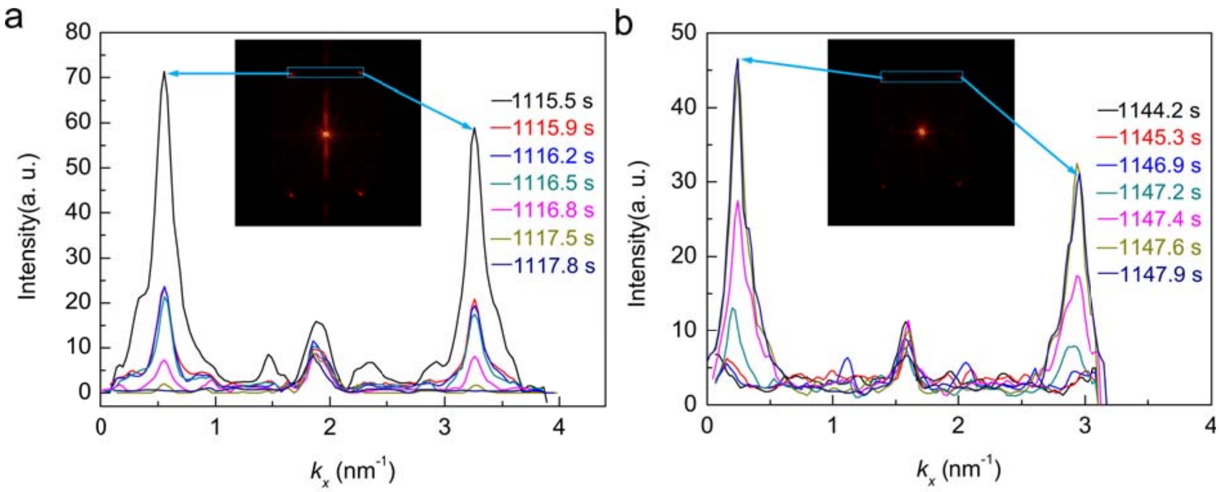

Supplementary Figure 9. a, b, The integrated profiles taken from the 2D FFT images in Fig. 2 and Fig. 3, respectively. The insets are two-dimensional FFT patterns processed in software WxSM. The integrated profiles were transformed from the boxed regions in **a** and **b** using the Fit2D program developed by Dr. Hammersley of the ESRF[1]. The peaks corresponding to the diffraction spots are indicated by the arrows.

Supplementary References

1. Hammersley, A. FIT2D V12.012 Reference Manual, v 6.0; ESRF: Grenoble, France; http://www.esrf.eu/computing/scientific/FIT2D/FIT2D_REF/fit2d_r.html.
